# Genomic imprinting: An epigenetic regulatory system

**DOI:** 10.1371/journal.pgen.1008970

**Published:** 2020-08-06

**Authors:** Marisa S. Bartolomei, Rebecca J. Oakey, Anton Wutz

**Affiliations:** 1 Epigenetics Institute, Department of Cell and Developmental Biology, Perelman School of Medicine, University of Pennsylvania, Philadelphia, Pennsylvania, United States of America; 2 Department of Medical & Molecular Genetics, King’s College London, London, United Kingdom; 3 D-BIOL, Institute of Molecular Health Sciences, Swiss Federal Institute of Technology, ETH Hönggerberg, Zurich, Switzerland

This series of articles on genomic imprinting and allele-specific expression in X chromosome inactivation honors Dr. Denise Barlow (1950–2017), who was a trail blazer in the field of genomic imprinting. Dr. Barlow was one of the first to identify imprinted genes, which are expressed and regulated in a parent-of-origin–specific manner, and among the first to establish mechanisms of coordinated regulation of imprinted genes in clusters.

Parental-specific chromosome behavior was noted in arthropods and marsupials more than 50 years ago. In mammals, inheritance patterns of observable phenotypes also suggested parent-of-origin–specific effects. In humans for example, cytological deletions of a small part of chromosome 15 had been associated with Prader–Willi and Angelman syndromes, whereby the paternally or maternally derived chromosome carried a deletion, respectively. Similarly, in mice classical geneticists generated and studied chromosome translocations to map genes. Some of these mouse strains showed parental-specific inheritance of phenotypes. From these studies the hairpin-tail mouse came to light, which carried a large deletion of chromosome 17 and demonstrated midgestation overgrowth and lethality when maternally transmitted. In contrast, paternal inheritance of the same deletion resulted in viable and fertile mice [1]. Dr. Barlow was insightful enough to seize upon these non-Mendelian patterns to develop the model for the career-long pursuit of gene regulatory mechanisms at this locus. These mice were critical reagents used by Dr. Barlow to clone *Igf2r*, one of the first identified imprinted genes [2]. Since that time, hundreds of imprinted genes have been identified, with the majority exhibiting conserved expression patterns among mammals.

Studies focusing on the regulation of imprinting were motivated by the observation that an active and inactive allele of a gene were present in the same nucleus and exposed to the same transcription factors but behaved differently. It became apparent that information along the DNA of the gene was responsible for “remembering” the parent of origin. Imprinted genes have many notable features that set them apart from the vast majority of the genome. First, imprinted genes exhibit parental-allele–specific DNA methylation at discrete elements, which is added in the germline and maintained through a phase of extensive reprogramming that occurs after fertilization in other parts of the genome. These elements are termed imprinting control regions (ICR) or imprinting control elements (ICE), as denoted by Barlow, and are critical for the appropriate allele-specific expression of adjacent gene(s). Barlow also was the first to describe secondary differentially methylated regions, which were acquired postfertilization, and are established as a consequence of imprinted gene expression. The discovery of DNA methylation at ICRs opened up the concept of DNA methylation acting as a widespread essential genomic regulatory device. In 1993, Denise Barlow proposed the novel idea that genomic imprinting might have arisen from a host defense mechanism designed to inactivate retrotransposons [3]. In this collection, Walsh and colleagues revisit this model and describe the machinery for acquisition and maintenance of DNA methylation at imprinted loci [4].

The vast majority of imprinted genes are located in clusters throughout the genome and are jointly regulated, typically through shared ICRs. Deletion of ICRs or perturbation of their allelic DNA-methylation patterns can cause loss of imprinting of multiple genes in *cis*. Key to understanding imprinting in many clusters is the presence of long noncoding (lnc) RNAs. Barlow and colleagues identified the first lncRNA at the *Igf2r* locus, *Airn*, whose greater than 100 kb transcript is initiated from the unmethylated ICR residing in an *Igf2r* intron. LncRNAs have multiple functions at imprinted (as well as other) loci. With respect to the *Igf2r* locus, many years of elegant experiments by the Barlow laboratory demonstrated that the lncRNA was not required for imprinting in the embryo proper but, rather, that *Airn* transcriptional overlap through the *Igf2r* promoter precludes RNA polymerase II recruitment [5]. MacDonald and Mann detail our current understanding of lncRNA functions through their transcription as well as their RNA product [6]. With respect to their RNA product, some lncRNAs are precursors of smaller RNAs or serve as scaffolds, guides, or architectural components. A recent investigation of *Airn* in regulating distant imprinted genes in mouse placenta rules out an enhancer and transcription interference-based mechanism [7]. This result points to distinct mechanisms regarding how *Airn* regulates the proximal *Igf2r* and more distant imprinted genes.

Early on, models that sought to explain why diploid mammals would support functional haploidy at imprinted genes suggested that these genes play important roles in growth of the fetus, in part balancing the conflicts between mother and father. It has become increasingly clear that imprinted genes have unique functions in the placenta, some genes of which are only expressed and/or imprinted in the placenta. Moreover, their regulation may differ from genes that are imprinted in the soma. In this collection, Courtney Hannah discusses the function and regulation of imprinted genes in the placenta, with special consideration given to the role of endogenous retroviruses (ERVs) in mediating placental-specific imprinting [8].

Because of the unusual nature of imprinting, the identification and study of imprinted genes have driven the adaption and modification of methods and, in some cases, necessitated the development of new technology. Denise Barlow embraced technology from the early days of positional cloning of genes, to the use of mouse knockout strategies for the study of regulatory elements and the requirement of lncRNAs, to the use of microarrays for identifying novel lncRNAs and characterizing chromatin structure at imprinted gene clusters. As described by Li and Li, the earliest studies of imprinting employed elegant embryological and genetic tools [9]. Initially, these tools were used to show the functional nonequivalence of the parental genomes and to map putative chromosomal locations of imprinted genes. Ultimately, the identification of imprinted genes relied on uniparental embryos and technologies that distinguish parental alleles in hybrid animals. More recently, high throughput technologies have facilitated the study of epigenetic processes and have benefitted from added read depth and the ability to study DNA modifications. Additionally, nuclear transplantation, haploid embryonic stem cells combined with site-directed deletions have more recently shown that the main block to uniparental embryo development is caused by imprinted gene expression.

Importantly, as the field of genomic imprinting matured, so did studies of X chromosome inactivation, a mechanism for mammals to achieve dosage compensation between females with two X chromosomes and males with one. In mice and marsupials, imprinted expression of the X chromosome was noted prior to the identification of imprinted genes. Although most mammals exhibit random X inactivation in somatic cells, paternal-specific inactivation of one of the two X chromosomes is observed in all cells of female marsupials and in mouse placentas. Due to overlap and similarities, including the role of the master regulator lncRNAs *Xist* and *Rsx*, investigators in these fields would learn from each other, often employing similar technologies and strategies to elucidate mechanisms. In this collection, Loda and Heard describe the role of *Xist* RNA and how it works to silence one X chromosome in *cis* [10].

Although genomic imprinting is itself a critical and fascinating topic, with important implications for human disease, Denise Barlow always argued that genomic imprinting was an influential model for mammalian epigenetic regulation. Insight gained from imprinted genes also helps to understand other important mechanisms of monoallelic expression, including immune and olfactory receptor gene expression, which is random rather than parent-of-origin specific in mammals. Given the need to maintain parental identity of imprinted genes from the gametes over many cell divisions in development, epigenetic mechanisms are essential for such processes. Although much has been learned, much remains to be determined in the imprinting field. Access to embryos at very early stages as well as technologies that facilitate single cell analysis will undoubtedly contribute to answering many remaining questions this field. The manuscripts in this series will provide historical perspective as well as insights from studying imprinting that have broad implications for biology. There is absolutely no doubt to the lasting legacy of Denise Barlow.
